# The Effect of Influencer Image Types on Purchase Intention: The Mediating Role of Persuasion Knowledge and the Moderating Role of Parasocial Relationships

**DOI:** 10.3390/bs16081270

**Published:** 2026-07-24

**Authors:** Yubing Qu, Jieun Lee

**Affiliations:** Department of Business Administration, Chung-Ang University, Seoul 06974, Republic of Korea; bingbing0901@naver.com

**Keywords:** influencer image type, persuasion knowledge, parasocial relationships, purchase intention, Instagram marketing, social media advertising

## Abstract

Understanding how visual content shapes consumers’ persuasion processes in social media environments has become an important research issue. Drawing primarily on the Persuasion Knowledge Model (PKM) and parasocial relationship (PSR) theory, while incorporating insights from self-reference theory and the Elaboration Likelihood Model (ELM), this study investigates how three Instagram image types—brand selfies, consumer selfies, and packshots—influence purchase intention through persuasion knowledge and examines the moderating role of PSR. A between-subjects experiment was conducted with 460 participants recruited through Amazon Mechanical Turk. The results revealed significant differences in persuasion knowledge and purchase intention across image types. Brand selfies were associated with lower levels of persuasion knowledge and higher purchase intention, whereas consumer selfies were associated with higher persuasion knowledge and lower purchase intention, with packshots showing intermediate effects. Persuasion knowledge mediated the relationship between image type and purchase intention, and PSR attenuated the negative effect of persuasion knowledge on purchase intention. These findings suggest that visual image composition itself can function as a persuasive cue and highlight the important role of relational context in shaping consumers’ responses to persuasive intent in influencer marketing.

## 1. Introduction

Visual content has become a central component of social media communication and brand interaction, particularly on image-oriented platforms such as Instagram. Compared with text-based media, visual content more effectively attracts attention, elicits emotional responses, and enhances user engagement (Hartmann et al., 2021). In social media environments, users actively seek entertainment, social interaction, self-expression, and identity management through online activities (Muntinga et al., 2011; Sheldon & Bryant, 2016), making visually rich content increasingly important in shaping consumer experiences. On Instagram, users frequently observe others’ daily lives while simultaneously presenting themselves through images, further reinforcing the central role of visual cues in social interaction and self-presentation. Prior research has shown that images containing faces or highly self-expressive elements can increase social presence and engagement (Bakhshi et al., 2014).

Influencers have become central actors in this ecosystem, serving as intermediaries between brands and consumers. Depending on their follower size and market reach, influencers are often classified as nano-, micro-, macro-, and mega-influencers, each differing in perceived authenticity, audience intimacy, and persuasive impact (C. Campbell & Farrell, 2020). Recent industry reports indicate that influencer marketing expenditures continue to expand, with many firms planning to increase their influencer marketing budgets in the coming year. Instagram remains one of the leading platforms for influencer-brand collaborations, accounting for 15% of platform selections in 2026 (Influencer Marketing Hub, 2026). These trends highlight the increasing strategic importance of understanding how influencer-generated visual content shapes consumer responses.

In this context, user-generated content (UGC) has emerged as a key form of brand communication. Consumers no longer merely consume brand-related content but also actively contribute to and create it, thereby forming ongoing relationships with brands. Muntinga et al. (2011) conceptualized these activities as consumers’ online brand-related activities (COBRAs), including consuming, contributing, and creating. These activities are driven by psychological motivations that encourage consumers to engage with brands while expressing their identities and experiences.

Among these user-generated forms of visual self-presentation, selfies have become one of the most prominent communication formats on social media. A selfie is more than a self-portrait; it is a visual communication format through which individuals construct relationships with products, spaces, and others. Through this process, brands become integrated into consumers’ everyday self-presentation. Previous research suggests that such humanized visual elements can make brands appear more relatable and emotionally engaging, thereby enhancing the effectiveness of brand communication (Sheldon & Bryant, 2016). In marketing contexts, purchase intention represents consumers’ subjective likelihood of purchasing a product and is widely regarded as a key indicator of persuasive effectiveness. Moreover, purchase intention is a central behavioral outcome in influencer marketing because it reflects consumers’ readiness to translate persuasive exposure into marketplace action.

Early studies classified Instagram images into broad content categories, such as selfies, food, fashion, activities, pets, and friends, demonstrating that users systematically share different types of visual content (Hu et al., 2014). Building on this line of research, subsequent studies shifted attention from general content categories to more specific visual presentation strategies used in influencer marketing. For example, Jin and Ryu (2020) investigated multiple Instagram photo types, including selfies, group photos, photos taken by others, product photos, and model photos, and demonstrated that variations in visual presentation significantly influence consumers’ parasocial interaction, envy, purchase intention, source trustworthiness, and brand trust.

More recently, Hartmann et al. (2021) proposed a more fine-grained typology of brand-related social media images into three categories: (1) packshots presenting only the product, (2) consumer selfies featuring both the consumer’s face and the product, and (3) brand selfies showing the product being held without revealing the face. Their findings indicated that consumer selfies generated higher engagement metrics, such as likes and comments, whereas brand selfies elicited relatively stronger purchase intentions. These findings suggest that commonly used engagement indicators on social media may not fully capture actual persuasive effectiveness.

Prior research has shown that the way products are visually presented influences consumers’ information processing and behavioral responses (Elder & Krishna, 2012). Building on self-reference theory (Rogers et al., 1977), encouraging consumers to imagine themselves using a product facilitates self-referential processing (Escalas, 2007). According to the Elaboration Likelihood Model (Petty & Cacioppo, 1986), message cues may induce either deeper elaboration or more heuristic processing depending on consumers’ motivation and ability to process information. Together, these theoretical perspectives provide an important foundation for understanding why different visual image types may produce different persuasive outcomes.

Whereas self-reference theory and Elaboration Likelihood Model (ELM) explain how visual cues shape information processing, the Persuasion Knowledge Model (PKM) provides additional insight into how consumers interpret persuasive intent in influencer-generated content and marketing messages more broadly. PKM refers to consumers’ accumulated knowledge about persuasion attempts, which they use to interpret and respond to persuasive messages (Friestad & Wright, 1994). When consumers recognize the commercial intent of a message, they are more likely to critically evaluate it, often leading to skepticism, resistance, and less favorable purchase-related responses (Boerman et al., 2012; M. C. Campbell & Kirmani, 2000; Eisend & Tarrahi, 2022). In influencer marketing contexts, this process becomes especially relevant because influencer content often blurs the boundary between everyday communication and commercial persuasion. As a result, consumers must infer persuasive intent from cues such as sponsorship disclosures and brand mentions, which may influence persuasion knowledge and message evaluations (Wojdynski, 2016; Yuan & Lou, 2020).

However, consumers do not interpret persuasive intent in isolation. Their responses to commercial messages may depend on the relational context in which those messages are encountered. In influencer marketing, consumers often develop parasocial relationships (PSRs), which refer to one-sided emotional bonds formed with media figures through repeated exposure and perceived interaction (Horton & Wohl, 1956; Tukachinsky & Stever, 2019). Prior research suggests that strong PSRs may reduce persuasion resistance and mitigate negative reactions to commercial content by increasing trust and perceived relational closeness (Breves et al., 2021; Hwang & Zhang, 2018). Thus, PSRs may shape how consumers interpret and respond to persuasive intent in influencer-generated content.

Although prior studies have separately identified the importance of image types in visual social media, the central role of persuasion knowledge, and the moderating role of PSRs, several important gaps remain. First, although previous studies have examined different image types in social media marketing (e.g., Hartmann et al., 2021) and persuasion knowledge in sponsored communication (e.g., Boerman et al., 2021; van Reijmersdal et al., 2022), limited research has examined whether different image types (i.e., brand selfies, consumer selfies, and packshots) are associated with different levels of persuasion knowledge. Second, although persuasion knowledge has been examined as a mediating mechanism (e.g., Brinson et al., 2024; Xu et al., 2026) and PSRs have been shown to shape consumers’ responses to influencers (e.g., Balaban et al., 2022; Boerman & van Reijmersdal, 2020), few studies have simultaneously investigated the mediating role of persuasion knowledge and the moderating role of PSR within a single integrated framework. Third, existing studies have primarily focused on engagement-based and behavioral outcomes, such as likes, comments, purchase intention, and brand trust (Hartmann et al., 2021; Jin & Ryu, 2020), with comparatively less attention given to differences in persuasive effectiveness across visual image types.

To address these gaps, the present study investigates how three image types commonly used in Instagram contexts influence consumers’ purchase intentions and how persuasion knowledge may help explain these relationships. Building on Hartmann et al. (2021), this study extends prior research by examining persuasion knowledge as a cognitive mechanism and PSRs as a relational context that may help explain how and when different image types shape purchase intention.

Specifically, this study proposes an integrated framework in which image type influences purchase intention through persuasion knowledge, while PSRs moderate this indirect effect. By doing so, the present study aims to provide a more nuanced understanding of social media image strategies and clarify how cognitive and relational factors jointly shape persuasion processes in influencer marketing contexts.

## 2. Literature Review and Hypothesis Development

### 2.1. Image Types, Self-Referential Processing, and Purchase Intention

Social media platforms enable users to create, share, and interact with content, thereby facilitating ongoing communication between consumers and brands (Kietzmann et al., 2011). Among these platforms, Instagram has emerged as a major channel for influencer marketing, where image-based communication plays a central role. Influencers continuously produce visual content that affects consumers’ attitudes and behaviors (Freberg et al., 2011), highlighting the importance of understanding how consumers interpret and respond to influencer-generated content. Moreover, recent research indicates that the effectiveness of influencer marketing depends substantially on the characteristics of the content itself, with content design playing a critical role in shaping consumers’ purchase intentions (Arora et al., 2020). In addition, content characteristics such as interactivity, informativeness, and entertainment have been shown to enhance consumers’ evaluations (De Battista et al., 2025), suggesting that the manner in which social media content is presented is an important determinant of consumer responses.

Given the visual nature of Instagram-based influencer communication, the way images are presented may play an important role in shaping consumer responses. Compared with textual information, visual content is often processed more intuitively and can evoke stronger emotional and cognitive responses (Bone & Ellen, 1992; Paivio, 1990). Recent studies further suggest that the informativeness, entertainment, and personalization of social media advertising enhance consumers’ perceived advertising value (Arora & Agarwal, 2019). In social media environments, visual executional elements such as images and photographs play a central role in shaping consumer attention and engagement (Casaló et al., 2020; Voorveld et al., 2018). Accordingly, differences in image composition may affect how consumers perceive and respond to influencer marketing content.

To explain these differences, this study draws on self-reference theory and the ELM (Petty & Cacioppo, 1986; Rogers et al., 1977). Self-reference theory suggests that individuals process information more deeply and favorably when it is connected to the self (Escalas, 2007; Rogers et al., 1977). In social media environments, visual content that promotes mental simulation and enhances personal relevance may therefore contribute to more favorable persuasion outcomes. Similarly, the ELM suggests that the allocation of attention between central product cues and peripheral social cues influences the depth of information processing and subsequent persuasion outcomes (Petty & Cacioppo, 1986).

Image perspective plays an important role in shaping self-referential processing. First-person visual perspectives encourage consumers to mentally simulate themselves using products and increase psychological proximity to the consumption experience (Elder & Krishna, 2012). Such immersive processing may contribute to more favorable behavioral responses, including purchase intention (Escalas, 2007; Yoo & Kim, 2014). By contrast, third-person perspectives may direct attention toward external actors rather than the self, thereby reducing immersive and self-referential processing.

Different image types used in influencer marketing may activate distinct information-processing patterns because visual perspectives direct consumers’ attention to different aspects of visual information, which subsequently influences their judgments and behavioral responses (Libby & Eibach, 2011; Zhang & Yang, 2015). Brand selfies present products from a first-person perspective without revealing the influencer’s face, allowing consumers to experience the product from the actor’s viewpoint and focus more directly on the product usage context (Elder & Krishna, 2012; Pink, 2015). Viewing products from a first-person perspective may encourage consumers to mentally simulate product use by creating a stronger sense of involvement in the consumption situation, thereby increasing self-referential processing (Libby & Eibach, 2011; Trope & Liberman, 2010). Because visual attention remains focused on the product-related cues rather than peripheral social cues, brand selfies may encourage greater elaboration of product information, consistent with the central route proposed by the ELM (Petty & Cacioppo, 1986).

In contrast, consumer selfies prominently display both the influencer and the product. Because human faces naturally capture visual attention (Bakhshi et al., 2014), consumers may allocate greater attention to the influencer than to the product itself. Compared with first-person perspectives, this observer-oriented perspective encourages consumers to process the image from an external viewpoint rather than imagining themselves using the product (Libby & Eibach, 2011; Pronin & Ross, 2006). As consumers devote less attention to the product usage experience, opportunities for self-referential processing and mental simulation may be reduced, making heuristic processing relatively more likely than systematic product-focused elaboration (Petty & Cacioppo, 1986; Trope & Liberman, 2010). Moreover, although consumer selfies may appear more personal and intimate, they may simultaneously heighten perceptions of commercialized self-presentation and persuasive intent (Audrezet et al., 2020), potentially making the persuasive nature of the message more salient.

Packshots represent a more traditional advertising format in which only the product is displayed without human cues. In digital environments, where consumers cannot physically inspect products before purchase, visual product presentation serves as an important source of product information (McCabe & Nowlis, 2003; Peck & Childers, 2003). Although packshots may lack the immersive quality of first-person brand selfies and do not depict the active hand–product interaction that provides meaningful product-use cues (Luangrath et al., 2020), the absence of salient human cues allows consumers to maintain attention on the product itself. Accordingly, packshots may occupy an intermediate position in terms of self-referential processing and purchase intention because they preserve product-focused attention while lacking the immersive product-use cues associated with brand selfies.

Taken together, self-reference theory and the ELM suggest that different image types influence consumers’ purchase intention by shaping product-focused attention, self-referential processing, and mental simulation (Libby & Eibach, 2011; Libby et al., 2011; Trope & Liberman, 2010). Specifically, brand selfie images are expected to elicit stronger purchase intention than both consumer selfie images and packshot images because they combine product-focused attention with active product interaction and a first-person perspective that facilitates mental simulation (Elder & Krishna, 2012). By contrast, consumer selfie images are expected to generate the lowest purchase intention because visual attention is more likely to be directed toward the influencer than the product itself (Bakhshi et al., 2014). Packshot images are expected to generate higher purchase intention than consumer selfie images because they maintain product-focused attention, although they are expected to be less effective than brand selfie images due to the absence of interactive product-use cues. Accordingly, systematic differences in purchase intention are expected across the three image types. Specifically, the following hypotheses are proposed:

**Hypothesis** **1a.** *Purchase intention will be higher for brand selfie images than for consumer selfie images.*

**Hypothesis** **1b.** *Purchase intention will be higher for brand selfie images than for packshot images.*

**Hypothesis** **1c.** *Purchase intention will be higher for packshot images than for consumer selfie images.*

### 2.2. Persuasion Knowledge as a Cognitive Mechanism

Prior research has examined persuasion knowledge as an important intervening mechanism linking advertising cues such as sponsorship disclosures and embedded promotional signals to consumers’ evaluative and behavioral responses (Boerman et al., 2017; van Reijmersdal et al., 2022; Wojdynski et al., 2018).

Beyond influencing self-referential processing and purchase intention, image types may also shape how consumers infer persuasive intent in social media contexts. The PKM explains that consumers develop knowledge about persuasion attempts through repeated exposure to marketing communication and use such knowledge to interpret and respond to persuasive messages (Friestad & Wright, 1994). Through prior experiences and observations, consumers learn to recognize persuasion tactics and infer marketers’ underlying intentions (Shrum et al., 2012).

Persuasion knowledge consists of both conceptual and evaluative dimensions. Conceptual persuasion knowledge refers to consumers’ recognition of persuasive intent and tactics, whereas evaluative persuasion knowledge reflects consumers’ critical and affective responses toward persuasive messages (Boerman et al., 2012; Ham et al., 2015). When consumers recognize persuasive intent, consumers are more likely to critically evaluate persuasive attempts, leading to greater skepticism and resistance toward advertising messages (M. C. Campbell & Kirmani, 2000; Isaac & Grayson, 2017). Persuasion knowledge enables consumers to interpret persuasive messages more critically and to question marketers’ underlying motives, which in turn reduces favorable evaluations of the message and the advertised product (M. C. Campbell & Kirmani, 2000; Friestad & Wright, 1994). Thus, persuasion knowledge functions as the cognitive mechanism through which marketing cues influence subsequent consumer evaluations and behavioural responses. Consistent with this reasoning, prior research has shown that heightened persuasion knowledge is associated with less favorable advertising evaluations, weaker brand attitudes, and lower purchase intentions (Boerman et al., 2017; van Reijmersdal et al., 2022). Accordingly, higher levels of persuasion knowledge are expected to reduce consumers’ purchase intention because recognizing persuasive intent promotes more critical evaluations of marketing messages and greater persuasion resistance.

In social media environments, persuasion knowledge is particularly relevant because commercial messages are often embedded within ordinary content. Rather than encountering clearly identifiable advertisements, consumers frequently infer persuasive intent from contextual and visual cues. Sponsorship disclosures, explicit brand mentions, and overt selling attempts increase consumers’ recognition of persuasive intent and are associated with higher levels of persuasion knowledge (Boerman et al., 2017; Wojdynski, 2016). By contrast, content that appears more natural and seamlessly integrated into everyday experiences may be associated with lower levels of persuasion knowledge (Boerman & van Reijmersdal, 2016; Wojdynski et al., 2018).

Different image formats may therefore influence not only consumers’ attention allocation but also how naturally commercial messages are interpreted within social media contexts. Prior research on visual perspective suggests that first-person images facilitate viewers’ immersion into the product-use context and encourage consumers to experience the presented situation from the actor’s perspective rather than as outside observers (Hur et al., 2020; Pink, 2015). Because consumers become more immersed in the consumption experience, they may focus less on identifying persuasive motives and more on the product-use experience itself. Because immersive and self-referential processing directs consumers’ attention toward the product-use experience rather than toward evaluating persuasive intent, image types that facilitate such processing may be associated with lower levels of persuasion knowledge.

Brand selfies present products from a first-person perspective and emphasize everyday product usage without explicitly highlighting the influencer as a persuasive source. Prior research has shown that first-person visual perspectives encourage consumers to imagine themselves experiencing the consumption situation and enhance psychological immersion in the presented context (Hur et al., 2020). Consequently, consumers may interpret such content as a more natural consumption experience, making brand selfies less likely to activate persuasion knowledge and therefore expected to elicit lower levels of persuasion knowledge than the other image formats.

As influencer marketing becomes increasingly commercialized, highly personalized consumer selfies may function not only as cues of relational closeness but also as signals of persuasive intent. Consumer selfies prominently display both the influencer and the endorsed product, making the persuasive source visually salient. Unlike first-person images, third-person visual perspectives encourage viewers to adopt a more observational perspective toward the presented situation rather than directly experiencing the consumption situation (Hur et al., 2020; Libby & Eibach, 2011). Consequently, consumers may allocate greater attention to the influencer as the persuasive source, increasing the likelihood of inferring commercial intent. Prior research suggests that consumers are more likely to activate persuasion knowledge when persuasive intent is explicitly signaled through advertising cues such as sponsorship disclosures (Boerman et al., 2017). Similarly, research on commercial signaling in embedded advertising contexts indicates that promotional cues integrated into user-generated content can heighten consumers’ recognition of persuasive intent (Wojdynski et al., 2018). Accordingly, consumer selfies are expected to activate higher levels of persuasion knowledge and elicit more critical evaluations of the message than other image types.

Packshots, which display only the product without human cues, represent a more traditional advertising format. Although consumers may more easily recognize the commercial nature of packshots, the absence of salient social cues may reduce the interpersonal recommendation signals present in consumer selfies. Accordingly, packshots may generate intermediate levels of persuasion knowledge relative to brand selfies and consumer selfies.

Collectively, these arguments suggest that image type influences consumers’ recognition of persuasive intent, which subsequently shapes purchase intention through persuasion knowledge. Accordingly, persuasion knowledge is expected to function as the underlying mediating mechanism linking image type to purchase intention. Specifically, image formats that appear more natural and less commercially explicit are expected to reduce persuasion knowledge, whereas image formats that make persuasive intent more salient are expected to increase persuasion knowledge, thereby lowering purchase intention. Based on this reasoning, the following hypothesis is proposed:

**Hypothesis** **2a.** *Image type will significantly influence persuasion knowledge. Specifically, brand selfies will be associated with lower levels of persuasion knowledge, consumer selfies with higher levels of persuasion knowledge, and packshots with intermediate levels.*

**Hypothesis** **2b.** *Persuasion knowledge will be negatively associated with purchase intention.*

**Hypothesis** **2c.** *Persuasion knowledge will mediate the relationship between image type and purchase intention.*

### 2.3. PSRs as a Relational Context in Influencer Marketing

In influencer marketing contexts, consumers often develop PSRs with influencers through repeated exposure and perceived interaction. PSRs refer to one-sided emotional bonds that media users form with media figures over time, creating a sense of familiarity, intimacy, and relational closeness (Horton & Wohl, 1956; Tukachinsky & Stever, 2019). As these interactions accumulate, influencers may come to be perceived as socially familiar and personally meaningful, resembling friends or acquaintances rather than merely functioning as information sources.

Social media environments facilitate the development of PSRs more effectively than traditional media contexts because influencers foster intimacy and accessibility through continuous content sharing and ongoing interactions with followers (Carter, 2016; Hudders & De Jans, 2022). Through repeated content consumption and perceived self-disclosure, users may experience a stronger sense of connection with influencers, making these relationships feel more socially meaningful (Bond, 2016). Compared with traditional celebrities, influencers are often perceived as more similar and accessible, which strengthens consumer identification and trust in them (C. Campbell & Farrell, 2020; Djafarova & Rushworth, 2017).

These social bonds influence how consumers process and respond to persuasive messages. Consumers with high levels of PSR tend to perceive influencers as more trustworthy information sources and assign greater source credibility to them (Chung & Cho, 2017; Ohanian, 1990). Consequently, they are more likely to form favorable evaluations of both the message and the associated brand (Djafarova & Rushworth, 2017). Strong PSRs are also associated with more positive brand attitudes and higher purchase intentions (Aw & Labrecque, 2020; Ferchaud et al., 2018; Reinikainen et al., 2020).

By contrast, consumers with low levels of PSR may be less likely to rely on a favorable relational interpretation when evaluating influencer-sponsored messages. Consequently, once persuasive intent is recognized and persuasion knowledge is activated, they are more likely to evaluate persuasive messages critically and engage in coping responses that undermine persuasive outcomes. Consistent with the PKM, research has shown that evaluative persuasion knowledge regarding influencer endorsements can trigger coping behaviors that subsequently reduce favorable persuasion outcomes, and that consumers’ coping with influencer endorsements is shaped by their parasocial relationships with influencers (Borchers et al., 2022). Accordingly, in the absence of a strong parasocial relationship, activated persuasion knowledge is more likely to elicit skeptical and resistant responses, thereby strengthening its negative effect on purchase intention.

Based on this reasoning, the present study proposes that image type influences purchase intention through persuasion knowledge, while PSR moderates this indirect effect. Specifically, when PSR is high, the negative effect of persuasion knowledge on purchase intention is expected to weaken, thereby attenuating the indirect effect of image type on purchase intention through persuasion knowledge.

**Hypothesis** **3.** *PSR will moderate the mediating effect of persuasion knowledge on the relationship between image type and purchase intention, such that the negative effect of persuasion knowledge on purchase intention will be weaker when PSR is high.*

### 2.4. The Conceptual Model

**Figure 1:**
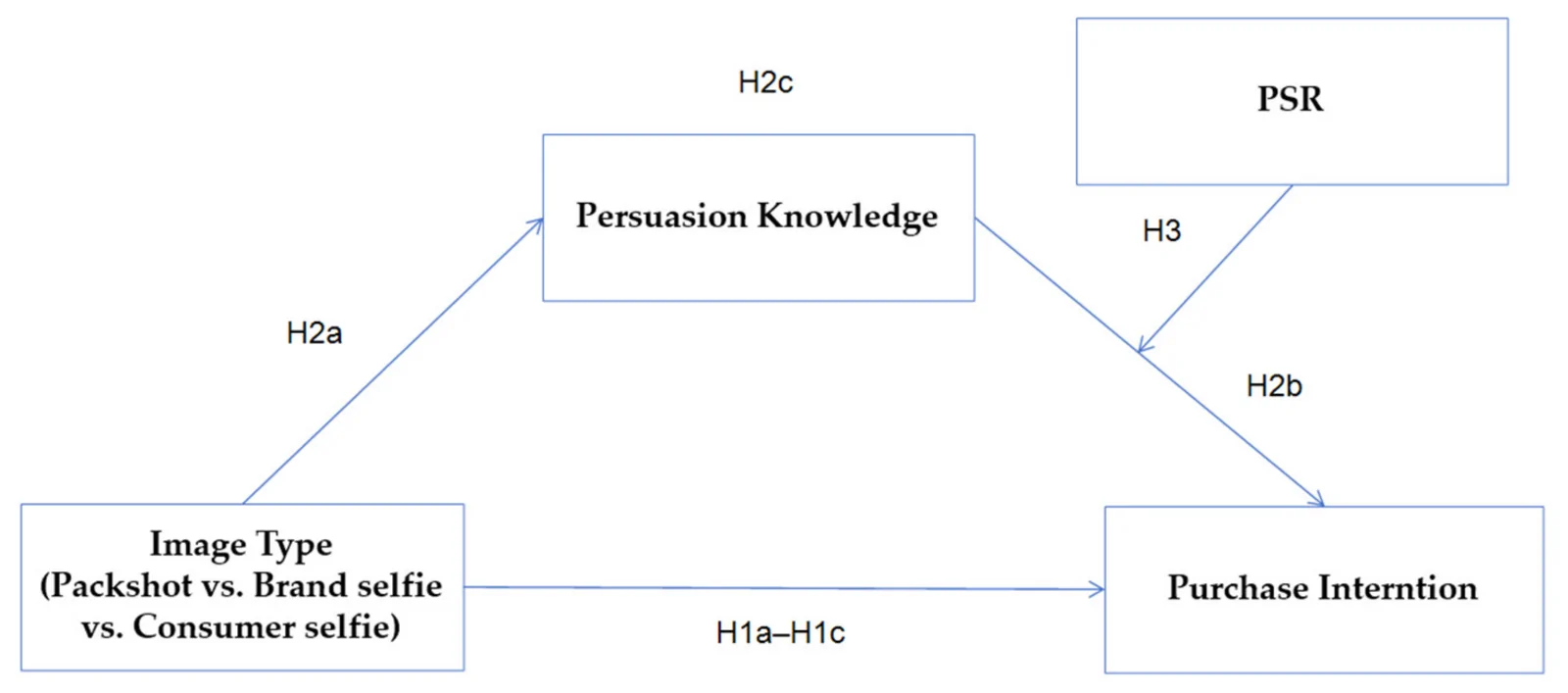
Conceptual model of hypothesized relationships.

Figure 1 presents the moderated mediation model proposed based on the above hypotheses.

## 3. Materials and Methods

### 3.1. Experimental Design and Stimuli

This study employed a one-factor between-subjects experimental design in which three Instagram post formats (brand selfie vs. consumer selfie vs. packshot) were manipulated as the independent variable. The experiment was designed to simulate Instagram, a representative image-centered social media platform where information is primarily communicated through visual content. Accordingly, all stimuli were created to resemble actual Instagram posts.

In the brand selfie condition, the influencer’s face was not shown, and the product was presented from a first-person visual perspective to highlight the product itself. In the consumer selfie condition, both the influencer’s face and the product were displayed together, making the connection between the endorser and the product explicit. In the packshot condition, a conventional product-centered image displaying only the product without any person was used.

Herbalife protein powder, a well-known brand in the health and wellness category, was selected as the stimulus product. This product category was considered appropriate because influencer marketing and social media-based recommendations are particularly prevalent in health and wellness contexts. The use of a widely recognized brand commonly encountered in influencer marketing enhanced the realism and ecological validity of the experimental stimuli.

To ensure visual consistency across conditions, the size and position of the product were kept constant across all images (Elder & Krishna, 2012), and the background was designed to be simple and neutral to minimize the influence of extraneous visual elements (see Appendix A).

### 3.2. Procedure

Prior to the main study, a pilot study was conducted with 90 participants (about 30 participants in each experimental condition) to evaluate the experimental procedure, questionnaire design, and measurement quality. Based on the pilot findings, minor refinements were made before the main data collection. A new independent sample was then recruited for the main study to ensure the independence of the final analyses.

The study was conducted online through Amazon Mechanical Turk (MTurk) in June 2024. Ethical procedures were followed throughout the study, including informed consent, voluntary participation, confidentiality, anonymity, and participants’ right to withdraw at any time. A total of 510 participants completed the survey, of whom 50 were excluded for failing the attention-check question, resulting in a final sample of 460 participants (48% female, 52% male; mean age = 34 years).

Three questionnaire versions corresponding to the three experimental conditions (brand selfie, consumer selfie, and packshot) were administered. Each participant completed only one version of the questionnaire and was exposed to a single image stimulus. Apart from the experimental manipulation (i.e., image type), all survey instructions, measurement items, and procedures were identical across the three conditions.

After providing informed consent, participants were exposed to an Instagram post uploaded by a fictitious influencer named “Kate,” who was developed specifically for this study. In the scenario, Kate was described as an influencer who shares fitness- and lifestyle-related content, and participants were instructed to imagine that they had been following the influencer for a long time. This setting was intended to establish a minimally plausible influencer-consumption context and a baseline sense of familiarity, allowing participants to meaningfully evaluate the content.

Following exposure to the stimulus, participants completed manipulation check items and then responded to measures assessing persuasion knowledge, PSR, and purchase intention. To control for factors that could influence the relationships among the focal variables, product familiarity, brand attitude, and purchase frequency of health-related products were included as covariates. Finally, demographic information such as gender and age was collected.

### 3.3. Measures

All major constructs were measured using established scales adapted from prior literature. All items were assessed using seven-point Likert-type scales ranging from 1 (strongly disagree) to 7 (strongly agree). Internal consistency reliability was evaluated using Cronbach’s alpha.

Manipulation check items were adapted from Hartmann et al. (2021) and modified for the context of the present study. These items were used to verify whether participants perceived the experimental manipulation as intended. Specifically, the manipulation check included items assessing attention to Herbalife protein powder and the prominence of Herbalife protein powder in the image. These items were used to examine whether the product received differential visual attention across the three image conditions.

Consistent with prior research, persuasion knowledge was operationalized as a broader evaluative-cognitive construct that captures both consumers’ recognition of persuasive intent and their critical or affective responses toward persuasive messages (Boerman et al., 2012; Ham et al., 2015). Accordingly, persuasion knowledge (mediator) was measured using seven items adapted from Vashisht and Royne (2016) and Hwang and Zhang (2018). A sample item is “The way this Instagram post image that Kate sent tries to persuade people seems acceptable to me.” The scale demonstrated high internal consistency reliability (Cronbach’s α = 0.92).

PSR (moderator) was assessed using a six-item scale adapted from Kim et al. (2015). The scale captures consumers’ perceived emotional connection and relationship with the influencer. A sample item is “I feel close enough to Kate to use her Instagram.” The scale demonstrated satisfactory reliability (Cronbach’s α = 0.89).

Purchase intention (dependent variable) was measured using three items adapted from Holzwarth et al. (2006). A sample item was: “I would like to try the Herbalife protein powder shown in the post image.” The scale exhibited acceptable reliability (Cronbach’s α = 0.85). The complete measurement items for all constructs are presented in Table 1.

In addition, product familiarity, attitude toward the brand, and purchase frequency of related products were included as covariates. The measurement items for these variables were adapted from Hartmann et al. (2021) and modified for the context of the present study.

### 3.4. Reliability and Validity Assessment

Because this study employed well-established reflective constructs, confirmatory factor analysis (CFA) using covariance-based structural equation modeling (CB-SEM) was conducted to validate the measurement model prior to hypothesis testing (Dash & Paul, 2021). CB-SEM was selected because the objective of the study was theory confirmation and measurement model validation rather than prediction or exploratory model development. Accordingly, IBM SPSS Amos, Version 29.0 (IBM Corp., Armonk, NY, USA) was used to conduct CFA to evaluate the reliability, convergent validity, discriminant validity, and overall model fit of the measurement model.

The overall model fit was evaluated using multiple goodness-of-fit indices. The measurement model demonstrated an excellent fit to the data. The chi-square value was 96.329 with 101 degrees of freedom (χ^2^/*df* = 0.954), well below the recommended threshold of 3.0. In addition, all fit indices exceeded the recommended criteria (GFI = 0.975, AGFI = 0.967, NFI = 0.977, IFI = 0.999, TLI = 0.999, CFI = 0.999, RMSEA = 0.000, and RMR = 0.057), indicating that the proposed measurement model adequately represented the observed data.

Table 1 presents the standardized factor loadings, Cronbach’s α, composite reliability (CR), and average variance extracted (AVE) for all constructs. All standardized factor loadings exceeded the recommended threshold of 0.70. Cronbach’s α values ranged from 0.85 to 0.92, while CR values ranged from 0.85 to 0.92, exceeding the recommended value of 0.70. Furthermore, all AVE values ranged from 0.56 to 0.66, exceeding the recommended threshold of 0.50. These findings indicate satisfactory internal consistency and convergent validity.

Discriminant validity was assessed using the Fornell–Larcker criterion (Fornell & Larcker, 1981). As shown in Table 2, the square root of the average variance extracted (AVE) for each construct exceeded the corresponding inter-construct correlations. Specifically, the square roots of AVE were 0.784 for persuasion knowledge, 0.750 for PSR, and 0.811 for purchase intention, all of which were greater than the absolute values of the correlations with the other constructs. Therefore, the results support satisfactory discriminant validity of the measurement model.

Table 3 reports the heterotrait–monotrait ratio (HTMT) values used to assess discriminant validity. All HTMT values ranged from 0.142 to 0.654, which are well below the conservative threshold of 0.85 (Henseler et al., 2015). These findings indicate that each construct is empirically distinct from the others, thereby supporting the discriminant validity of the measurement model. Overall, the CFA results, reliability analysis, convergent validity, and discriminant validity tests consistently support the adequacy of the measurement model. Therefore, the measurement model was considered appropriate for testing the proposed structural model.

To assess the potential influence of common method variance (CMV), Harman’s single-factor test (Harman, 1976) was conducted using an unrotated principal component analysis. As shown in Table 4. Prior to the factor analysis, the Kaiser–Meyer–Olkin (KMO) measure of sampling adequacy (Kaiser, 1974) was 0.928, indicating excellent sampling adequacy, and Bartlett’s test of sphericity (Bartlett, 1954) was significant (χ^2^ = 4123.019, *df* = 120, *p* < 0.001), confirming that the data were suitable for factor analysis. Three factors with eigenvalues greater than 1.0 were extracted, and the first unrotated factor explained 38.721% of the total variance, which is below the recommended threshold of 50% (Podsakoff et al., 2003). Therefore, the measurement properties of the constructs were considered satisfactory for the subsequent analyses.

## 4. Results

### 4.1. Manipulation Checks

To verify whether product centrality was perceived differently across image conditions, manipulation checks assessing attention to the Herbalife protein powder and the prominence of the Herbalife protein powder were performed.

First, a significant difference emerged across conditions in terms of the extent to which the product attracted participants’ attention (*F*(2, 457) = 5.85, *p* = 0.003). Mean comparisons indicated that the brand selfie condition (*M* = 5.75) and the packshot condition (*M* = 5.62) generated higher levels of product attention than the consumer selfie condition (*M* = 5.31).

Second, perceived product prominence also differed significantly across conditions (*F*(2, 457) = 6.62, *p* = 0.001). Participants in the brand selfie condition (*M* = 5.78) and the packshot condition (*M* = 5.70) perceived the product as more prominent than those in the consumer selfie condition (*M* = 5.34).

Taken together, the manipulation checks yielded consistent results, indicating that the consumer selfie condition generated relatively lower levels of product attention and product prominence. This finding suggests that the influencer’s face functioned as a stronger competing visual cue in the consumer selfie condition, thereby reducing the relative centrality of the product and confirming that the image-type manipulation operated in the intended direction. Descriptive statistics for key variables are presented in Table 5.

### 4.2. Statistical Assumptions and Preliminary Analyses

Before hypothesis testing, the assumptions underlying the ANOVA were evaluated. Because each experimental group had more than 150 participants (*N*_Packshot_ = 152, *N*_Brand selfie_ = 153, *N*_Consumer selfie_ = 155), the normality assumption was considered sufficiently satisfied based on the Central Limit Theorem. Given the large and approximately equal group sizes, ANOVA is generally robust to moderate violations of normality. Therefore, formal normality tests (e.g., Shapiro–Wilk or Kolmogorov–Smirnov) were not conducted, as such tests are highly sensitive to large sample sizes and may detect trivial deviations from normality that have limited practical implications for ANOVA (Field, 2024; Ghasemi & Zahediasl, 2012). Homogeneity of variance was assessed using Levene’s test (Levene, 1960). When the homogeneity assumption was violated, Welch’s robust ANOVA (Welch, 1951) was additionally performed to verify that the statistical conclusions remained robust under unequal variances. The results of Levene’s test and Welch’s robust ANOVA are presented in Table 6.

Levene’s tests (Levene, 1960) indicated that the assumption of homogeneity of variance was violated for both persuasion knowledge and purchase intention (*ps* < 0.01). Accordingly, Welch’s robust ANOVA (Welch, 1951), which does not assume homogeneity of variances, was conducted to verify whether the observed group differences remained significant despite the violation of the homogeneity assumption. The results remained significant for both variables (*ps* < 0.001), suggesting that the observed group differences were robust despite unequal variances.

### 4.3. Main Effect of Image Type on Purchase Intention

One-way ANOVA was employed to compare mean differences among the three experimental conditions because the independent variable consisted of three categorical groups and the dependent variables were measured on continuous scales. Image type was coded as a nominal variable with three categories (brand selfie, packshot, and consumer selfie).

**Figure 2:**
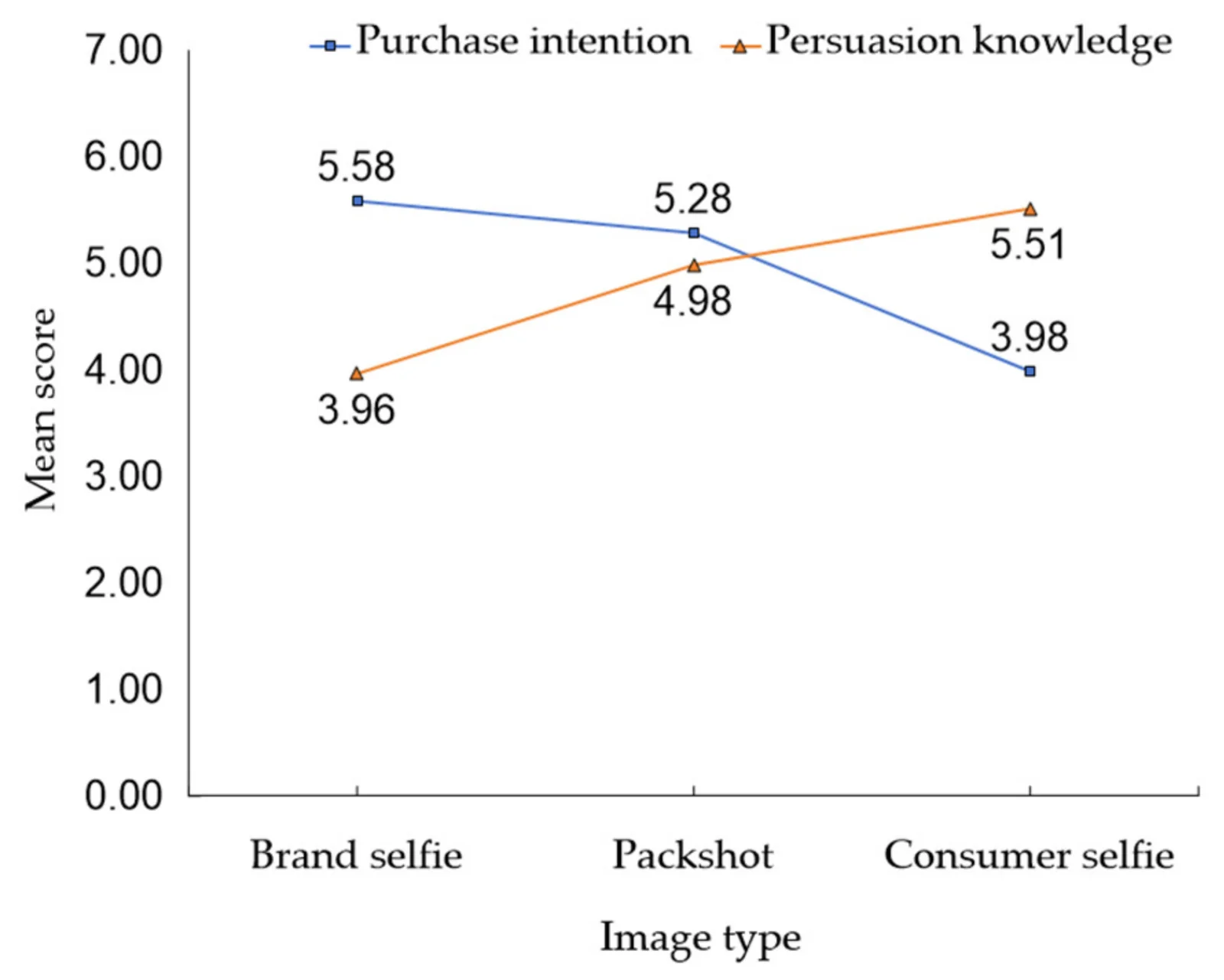
Differences in persuasion knowledge and purchase intention across image types.

The results revealed a significant main effect of image type on purchase intention (*F*(2, 457) = 68.16, *p* < 0.001). Mean comparisons indicated that purchase intention was highest in the brand selfie condition (*M* = 5.58, *SD* = 1.04), followed by the packshot condition (*M* = 5.28, *SD* = 1.25), whereas the consumer selfie condition showed the lowest level of purchase intention (*M* = 3.98, *SD* = 1.50) (see Figure 2).

Post hoc comparisons showed that purchase intention was significantly higher for the brand selfie condition than for the consumer selfie condition (Mean difference = 1.59, *p* < 0.001), supporting Hypothesis 1a. However, although purchase intention was descriptively higher in the brand selfie condition than in the packshot condition (Mean difference = 0.29), this difference was not statistically significant (*p* = 0.134). Therefore, Hypothesis 1b was not supported. In addition, purchase intention was significantly higher for the packshot condition than for the consumer selfie condition (Mean difference = 1.30, *p* < 0.001), supporting Hypothesis 1c.

Overall, these findings indicate that consumer selfie images generated significantly lower purchase intention than both brand selfie and packshot images, whereas purchase intention did not differ significantly between the brand selfie and packshot conditions.

### 4.4. Mediating Effect of Persuasion Knowledge

To examine the mediating role of persuasion knowledge, a mediation analysis was conducted using IBM SPSS Statistics (Version 22) with the PROCESS macro (Version 4.2) Model 4 (Hayes, 2017) with 5000 bootstrap samples. Image type was specified as a multicategorical independent variable, with the packshot condition serving as the reference category. Persuasion knowledge was entered as the mediator and purchase intention as the dependent variable. Product familiarity, brand attitude, and purchase frequency were included as covariates.

The results indicated that image type significantly predicted persuasion knowledge. Specifically, the brand selfie condition (relative to the packshot condition) resulted in significantly lower levels of persuasion knowledge (*b* = −1.02, *SE* = 0.13, *p* < 0.001), whereas the consumer selfie condition (relative to the packshot condition) resulted in significantly higher levels of persuasion knowledge (*b* = 0.53, *SE* = 0.13, *p* < 0.001). These findings support Hypothesis 2a, indicating that image type significantly influenced persuasion knowledge. As hypothesized, brand selfies elicited the lowest persuasion knowledge, consumer selfies the highest, and packshots intermediate levels.

Persuasion knowledge, in turn, negatively predicted purchase intention (*b* = −0.53, *SE* = 0.05, *p* < 0.001), supporting Hypothesis 2b. The mediation model demonstrated satisfactory explanatory power. The model predicting persuasion knowledge was significant (*R*^2^ = 0.238, *F*(5, 454) = 28.30, *p* < 0.001), explaining 23.8% of the variance. Likewise, the model predicting purchase intention was significant (*R*^2^ = 0.435, *F*(6, 453) = 58.08, *p* < 0.001), explaining 43.5% of the variance.

Bootstrap analyses further revealed significant indirect effects, supporting Hypothesis 2c. The bootstrap confidence intervals for both comparisons excluded zero, indicating that persuasion knowledge significantly mediated the relationship between image type and purchase intention. Specifically, the brand selfie condition (relative to the packshot condition) showed a significant positive indirect effect on purchase intention through reduced persuasion knowledge (ab = 0.55, BootSE = 0.09, 95% CI [0.38, 0.74]). In contrast, the consumer selfie condition (relative to the packshot condition) produced a significant negative indirect effect on purchase intention through increased persuasion knowledge (ab = −0.28, BootSE = 0.07, 95% CI [−0.42, −0.15]). The opposite directions of the indirect effects were consistent with the theoretical expectation that image types differentially influence persuasion knowledge, which in turn shapes purchase intention.

Regarding the covariates, brand familiarity positively predicted purchase intention (*b* = 0.12, *SE* = 0.03, *p* < 0.001). In contrast, attitude toward the brand (*b* = 0.02, *SE* = 0.03, *p* = 0.556) and frequency of health supplement purchases (*b* = 0.04, *SE* = 0.07, *p* = 0.496) were not significant predictors.

Taken together, these findings support Hypotheses 2a–c, demonstrating that persuasion knowledge significantly mediated the relationship between image type and purchase intention. Image type influenced persuasion knowledge, which in turn negatively predicted purchase intention, resulting in significant indirect effects across image-type conditions.

### 4.5. Moderated Mediation by PSR

To examine the moderated mediation effect of PSRs, an analysis was conducted using PROCESS Model 14 (Hayes, 2017) with 5000 bootstrap samples. Image type was dummy coded using the packshot condition as the reference category and specified as the independent variable, persuasion knowledge as the mediator, and purchase intention as the dependent variable. PSR was entered as a moderator of the relationship between persuasion knowledge and purchase intention.

The results showed that persuasion knowledge exerted a significant negative effect on purchase intention (*b* = −0.50, *SE* = 0.04, *p* < 0.001), whereas PSR had a significant positive effect on purchase intention (*b* = 0.32, *SE* = 0.04, *p* < 0.001). In addition, the interaction effect between persuasion knowledge and PSR was significant (*b* = 0.18, *SE* = 0.03, *p* < 0.001), indicating that the effect of persuasion knowledge on purchase intention varied depending on the level of PSR. The increase in explained variance resulting from the inclusion of the interaction term was also significant (Δ*R*^2^ = 0.034, *F*(1, 454) = 33.77, *p* < 0.001). The moderated mediation model also demonstrated satisfactory explanatory power. The model predicting persuasion knowledge was significant (*R*^2^ = 0.238, *F*(5, 454) = 28.30, *p* < 0.001), whereas the model predicting purchase intention was also significant (*R*^2^ = 0.552, *F*(8, 451) = 69.33, *p* < 0.001), explaining 55.2% of the variance in purchase intention.

**Figure 3:**
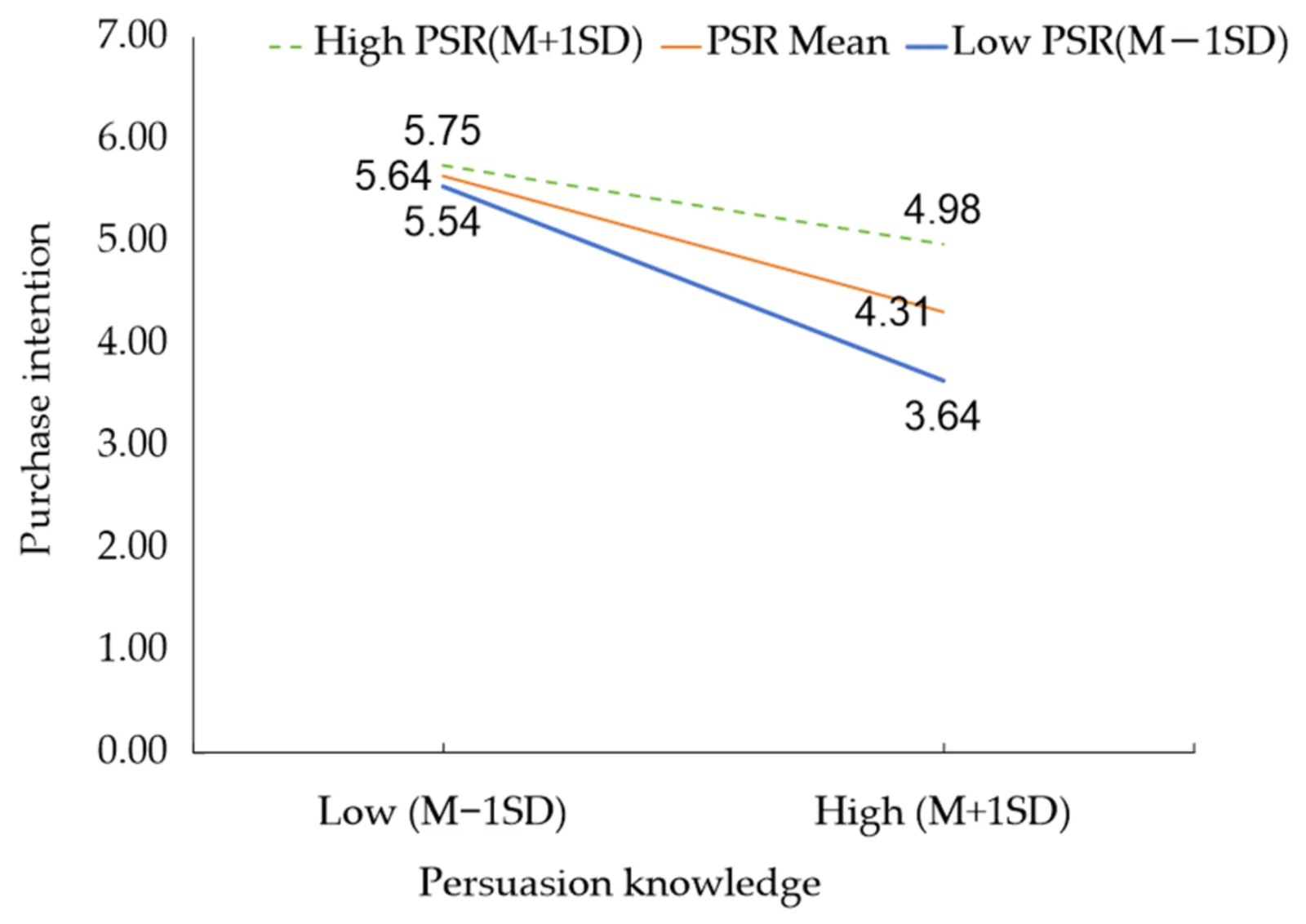
Interaction effect of persuasion knowledge and PSR on purchase intention.

Conditional effects were probed at low (−1 *SD*), mean, and high (+1 *SD*) levels of PSR using a spotlight analysis (Spiller et al., 2013). The results showed that the negative effect of persuasion knowledge on purchase intention was strongest at low levels of PSR (−1 *SD*; *b* = −0.71, *SE* = 0.05, *p* < 0.001), weaker at the mean level of PSR (*b* = −0.50, *SE* = 0.04, *p* < 0.001), and weakest at high levels of PSR (+1 *SD*; *b* = −0.29, *SE* = 0.05, *p* < 0.001). Floodlight analyses were conducted using the Johnson–Neyman technique (Spiller et al., 2013). The results indicated that no Johnson–Neyman transition point was identified within the observed range of PSR, meaning that the negative effect of persuasion knowledge on purchase intention remained statistically significant across the entire observed range of the moderator. These findings indicate that the negative effect of persuasion knowledge on purchase intention weakened as PSR increased, although it remained significant across the observed range of the moderator (see Figure 3).

**Figure 4:**
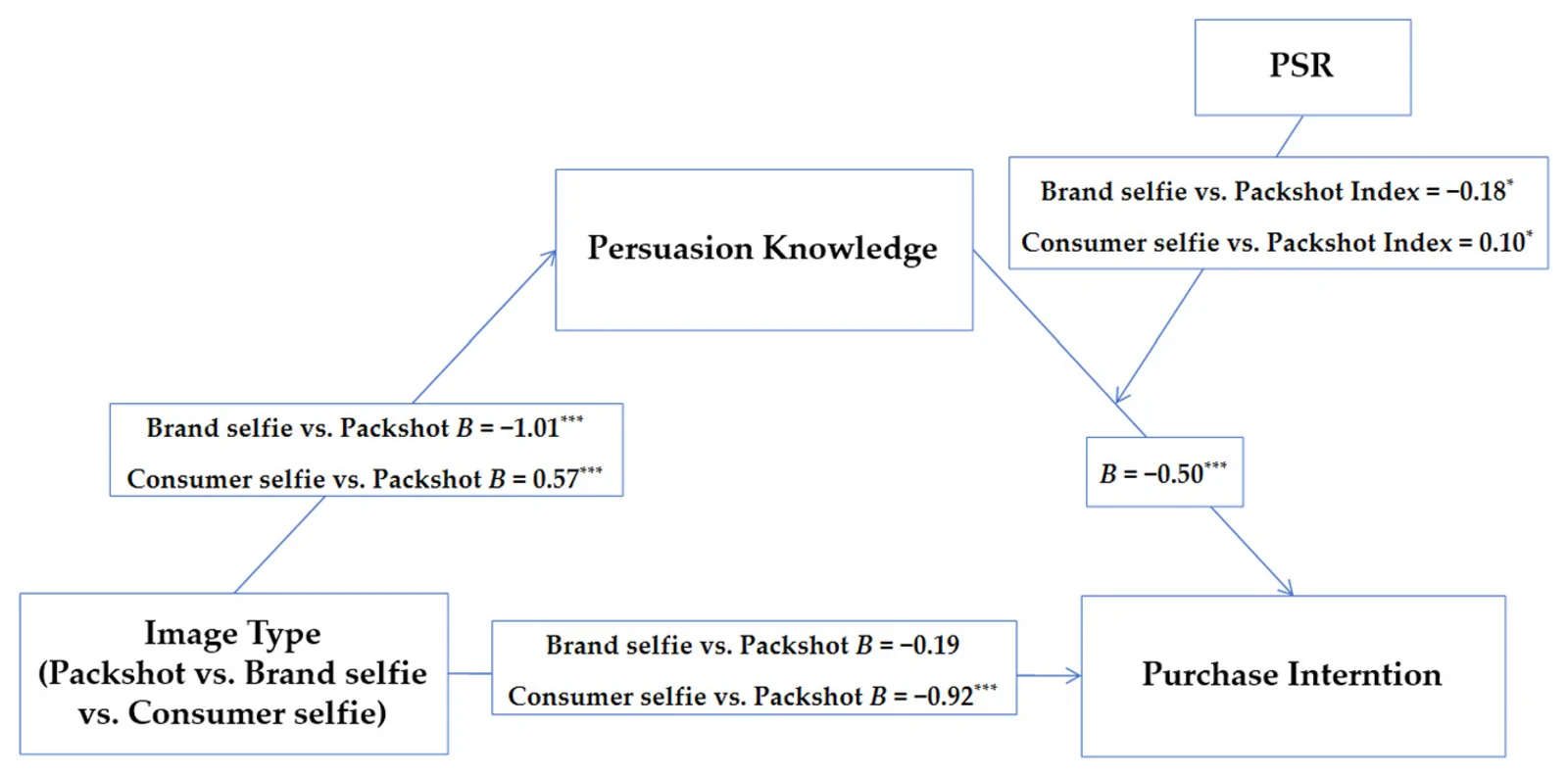
Moderated mediation model of the effects of image type on purchase intention through persuasion knowledge, moderated by PSR (* *p* < 0.05, *** *p* < 0.001).

The moderated mediation analysis further showed that, for the brand selfie condition (relative to the packshot condition), the index of moderated mediation was significantly negative (Index = −0.18, BootSE = 0.04, 95% CI [−0.26, −0.10]). For the consumer selfie condition (relative to the packshot condition), the index of moderated mediation was significantly positive (Index = 0.10, BootSE = 0.03, 95% CI [0.05, 0.16]). These results suggest that higher levels of PSR were associated with weaker indirect effects through persuasion knowledge, rather than changing the direction of those indirect effects (see Figure 4). Regarding the covariates, none of the control variables significantly predicted purchase intention after PSR and the interaction term was included in the moderated mediation model (brand familiarity: *b* = 0.05, *SE* = 0.03, *p* = 0.079; attitude toward the brand: *b* = −0.00, *SE* = 0.03, *p* = 0.882; frequency of health supplement purchases: *b* = 0.07, *SE* = 0.06, *p* = 0.239). These findings indicate that the moderated mediation effect remained robust after controlling for these variables.

**Figure 5:**
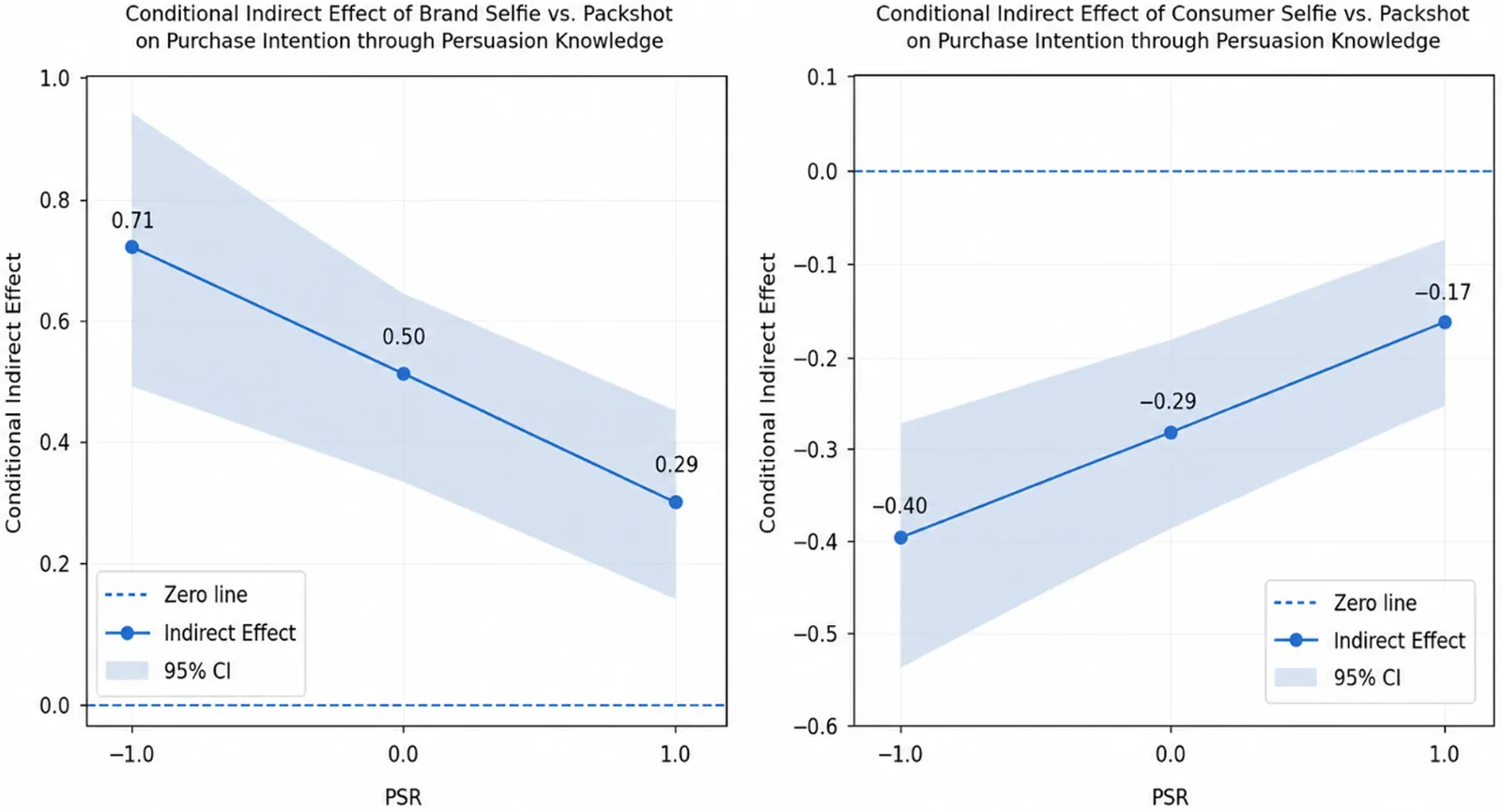
Conditional indirect effects of brand selfie and consumer selfie images (relative to packshots) on purchase intention through persuasion knowledge across levels of PSR.

Overall, the results support Hypothesis 3, demonstrating that PSR moderated the indirect effect of image type on purchase intention through persuasion knowledge. Specifically, the conditional indirect effect of brand selfie images relative to packshots was strongest at low levels of PSR (Effect = 0.71, 95% CI [0.492, 0.966]) and became weaker at high levels of PSR (Effect = 0.29, 95% CI [0.167, 0.445]). Likewise, the conditional indirect effect of consumer selfie images relative to packshots decreased from low PSR (Effect = −0.40, 95% CI [−0.608, −0.208]) to high PSR (Effect = −0.17, 95% CI [−0.277, −0.075]). These findings indicate that the indirect effect of image type on purchase intention through persuasion knowledge became weaker as PSR increased (see Figure 5).

Table 7 summarizes the proposed hypotheses, the relationships tested, the corresponding empirical findings, and the results of the hypothesis tests.

## 5. Discussion

### 5.1. Summary and Interpretation of Findings

This study examined how influencer image types shape consumers’ persuasion processes and purchase intentions in social media contexts. Specifically, it investigated the effects of brand selfies, consumer selfies, and packshots while identifying the mediating role of persuasion knowledge and the moderating role of PSR. The findings were largely consistent with the theoretical expectations.

First, brand selfies generated higher purchase intentions than consumer selfies, while packshots also produced higher purchase intentions than consumer selfies. However, the difference between brand selfies and packshots was not statistically significant. These findings are generally consistent with ELM and self-reference theory. The manipulation check results showed that brand selfies directed greater visual attention toward the product, whereas consumer selfies drew relatively more attention to the influencer, suggesting that image composition shapes how consumers allocate attention between the product and the communicator. In brand selfies, the first-person visual perspective may create attentional conditions conducive to mental simulation and self-referential processing (Elder & Krishna, 2012; Escalas, 2007), thereby contributing to more favorable purchase intentions. By contrast, the influencer’s face in consumer selfies may function as a salient visual cue that diverts attention away from the product and toward the communicator (Smith et al., 2018; To & Patrick, 2021), reducing the conditions under which self-referential processing may occur.

The lack of a significant difference between brand selfies and packshots suggests that packshots may also maintain product-focused attention and produce persuasive outcomes comparable to those of brand selfies (MacInnis & Jaworski, 1989). This null result raises important questions about the distinctiveness of first-person visual perspective as a unique persuasive mechanism. Although self-reference theory provides a plausible explanation for brand selfie effects, the comparable purchase intentions observed in the packshot condition suggest that self-referential processing may be only one of several pathways through which persuasive effectiveness is achieved. The effects of image type therefore appear to be shaped not simply by the presence of a human figure, but by the interplay between product-focused attention and self-referential processing. In addition, the effects of image type may vary depending on product category or consumer involvement, indicating the need for future research to examine broader situational and boundary conditions.

Second, persuasion knowledge varied systematically across image types, with brand selfies producing the lowest level, consumer selfies the highest, and packshots falling between the two conditions. Thus, the predicted rank-order pattern emerged for persuasion knowledge. Overall, the results extend the core logic of PKM—that consumers generate critical evaluations when they recognize persuasive intent (M. C. Campbell & Kirmani, 2000; Friestad & Wright, 1994)—to visually oriented social media content. Whereas prior PKM research has primarily focused on explicit verbal persuasion cues, such as sponsorship disclosures or advertising language, the present study suggests that visual image composition may also shape persuasion knowledge through consumers’ inferences about persuasive intent. However, because brand selfies and packshots did not differ significantly in purchase intention despite their differences in persuasion knowledge, this mechanism may not fully account for all image-type differences in purchase intention. This finding suggests that although persuasion knowledge is an important explanatory mechanism, additional cognitive or perceptual processes may also contribute to consumers’ purchase responses.

Finally, PSR significantly moderated the relationship between persuasion knowledge and purchase intention. Specifically, the negative effect of persuasion knowledge gradually weakened as the level of PSR increased. Floodlight analyses further confirmed that although the negative effect of persuasion knowledge remained significant across the entire range of PSR, its magnitude decreased as PSR became stronger. These findings suggest that PSR buffers the negative consequences of persuasion knowledge through relational trust and perceived intimacy (Breves et al., 2021; Hwang & Zhang, 2018). Accordingly, the influence of persuasion knowledge does not appear to operate as a universally applicable cognitive resistance mechanism, but instead varies depending on the relational context between consumers and influencers. In other words, even when consumers recognize persuasive intent, those who have formed strong PSRs with influencers may interpret persuasive messages more favorably and consequently form less negative evaluations. However, because PSR did not completely eliminate the negative effect of persuasion knowledge, the results also imply that influencer marketing strategies should focus not only on relationship building but also on maintaining the authenticity and naturalness of content.

### 5.2. Theoretical Implications

This study offers several theoretical implications for the literature on persuasion knowledge, visual social media content, and PSRs.

First, this study broadens the scope of the PKM by extending its application to visually oriented social media environments. Prior PKM research has mainly conceptualized persuasion knowledge as a response to explicit verbal persuasion cues, such as sponsorship disclosures, advertising claims, or brand mentions (Boerman et al., 2017). The present study suggests that visual image composition itself may also function as an antecedent of persuasion knowledge by shaping consumers’ inferences about persuasive intent. This expands the PKM framework beyond linguistic and disclosure-based cues and highlights the role of visual structure as a meaningful source of consumers’ inferences about persuasive intent in social media contexts.

Second, this study contributes to the literature on social media image strategies by shifting the focus from outcome-based comparisons to process-based explanations. Existing studies on image types have largely emphasized engagement metrics and behavioral outcomes (Hartmann et al., 2021), offering limited insight into the psychological processes underlying these effects. The present findings suggest that the effectiveness of image formats may depend not only on aesthetic preference or social presence, but also on how visual configurations shape attentional focus and subsequent interpretations of persuasive intent. By integrating self-reference theory, ELM, and PKM, this study provides a more theoretically grounded explanation of why different image formats may produce different persuasion outcomes.

Third, this study reconceptualizes PSRs as an important relational boundary condition in persuasion processes. Previous research has primarily treated PSR as a direct driver of favorable consumer responses through trust, intimacy, and identification (Breves et al., 2019; Labrecque, 2014). The present findings suggest that PSR also plays a more nuanced role by weakening the negative downstream effects of persuasion knowledge. This perspective extends the understanding of persuasion knowledge by showing that its consequences do not always operate in the same way, but instead vary depending on consumers’ relational bonds with influencers. Taken together, these findings integrate insights from PKM and PSR and offer a more relationship-sensitive explanation of how consumers respond to persuasive intent in influencer marketing environments.

### 5.3. Managerial Implications

The findings provide several managerial implications for influencer marketing practitioners.

First, image format should be managed not merely as a visual element but as a strategic variable that shapes consumers’ perceptions of persuasive intent and purchase intentions. The findings suggest that brand selfie formats may be particularly effective because they direct greater product-focused attention while eliciting relatively lower levels of persuasion knowledge. This implies that integrating products into natural, first-person usage contexts may help reduce persuasion resistance and create conditions that support more favorable consumer responses. Accordingly, when designing influencer content, brands should consider not only whether products are displayed, but also how different image formats may shape consumers’ attentional focus and perceptions of persuasive intent.

Second, engagement metrics and persuasion effectiveness may not necessarily align. Consumer selfies, which prominently display influencers’ faces, may attract greater attention and engagement responses (e.g., likes and comments), but at the same time may generate higher levels of persuasion knowledge, thereby limiting their effectiveness in generating purchase intentions. This finding suggests that high engagement does not necessarily translate into stronger persuasive outcomes. This may occur because consumer selfies rely primarily on socially salient cues, such as the influencer’s face, which can increase visibility and interaction while simultaneously making the persuasive intent of the content more salient. As a result, engagement-driven attention may not always lead to stronger purchase motivation. Therefore, practitioners should evaluate content performance not only through engagement metrics but also through behavior-oriented indicators such as purchase intention, brand attitude, and conversion rates.

Third, influencer selection should place greater emphasis on the quality of the relationship between influencers and followers rather than relying solely on follower size. The findings indicate that the negative effect of persuasion knowledge weakened as the level of PSR increased. This suggests that influencers who have established long-term and authentic relationships with followers may be more effective in reducing consumers’ defensive reactions triggered by persuasive intent. In particular, repeated sharing of everyday content and authentic interactions may encourage consumers to interpret commercial messages more favorably. However, because PSR did not completely eliminate the negative impact of persuasion knowledge, brands should focus not only on relationship building but also on maintaining the naturalness and authenticity of sponsored content. Accordingly, influencer strategies based on long-term partnerships and relational development may be more effective than campaigns focused solely on short-term exposure effects.

### 5.4. Limitations and Future Research Directions

Despite the theoretical and practical contributions of this study, several limitations should be acknowledged. These limitations relate primarily to the conceptualization and measurement of the focal constructs, the methodological choices, and the generalizability of the findings beyond the specific context examined in this study.

The first limitation concerns the conceptualization and measurement of the focal constructs. Specifically, this study treated persuasion knowledge as a unidimensional construct, despite prior literature distinguishing conceptual and evaluative dimensions of persuasion knowledge (Boerman et al., 2012; Ham et al., 2015). Although supplementary confirmatory factor analyses supported the adequacy and parsimony of the one-factor structure in the present sample, future research may further investigate whether different image types differentially influence specific dimensions of persuasion knowledge.

In addition, although product-focused attention was assessed as a manipulation check, self-referential processing and mental simulation were not directly measured in the present study. Consequently, the proposed cognitive mechanisms underlying image-type effects remain inferential rather than directly observed. Future research should incorporate direct measures of these processes to further clarify the psychological pathways linking image characteristics and persuasion outcomes.

The second limitation concerns the methodological choices adopted in this study. While these design decisions improved realism and reflected common social media consumption contexts, they may nevertheless limit the broader applicability of the findings.

First, this study relied on a single stimulus exemplar for each image-type condition and focused on a single product category within the health and wellness domain using a real and recognizable brand (Herbalife). The use of a single stimulus per condition makes it difficult to fully disentangle image-type effects from stimulus-specific characteristics, while the use of a real brand may have activated pre-existing brand associations and attitudes. Although the use of a well-known brand increased realism and reflected common influencer marketing practices, it may also have introduced systematic variation associated with participants’ prior familiarity with and attitudes toward the brand. While product familiarity, brand attitude, and purchase frequency were statistically controlled, such controls cannot fully eliminate these potential confounds. Future studies should employ multiple stimuli, model stimuli as random factors, examine broader product categories, and use fictitious or experimentally neutral brands.

Second, PSR was measured within a hypothetical scenario involving a fictitious influencer and a baseline familiarity instruction. These design choices were intended to establish a minimally plausible influencer-consumption context and enable meaningful evaluations of influencer content. However, they may not fully capture the naturally evolving nature of PSRs formed through repeated real-world exposure. Future studies may benefit from using actual follower–influencer relationships, longitudinal designs, or comparison conditions without familiarity primes.

Third, this study focused exclusively on static image-based Instagram content. Although static images remain common in influencer marketing, contemporary social media environments increasingly involve short-form videos, live commerce, and interactive content formats. Future research should examine whether the present findings generalize to these more dynamic content environments.

Finally, because the manipulation check items were administered before the main dependent measures, they may have increased participants’ attention to the product and influenced subsequent purchase intention responses. Although this ordering allowed us to verify the effectiveness of the experimental manipulations before assessing the substantive outcomes, it may also have introduced unintended carryover effects by increasing product salience. Future studies should consider administering manipulation checks after the main dependent measures to minimize such effects.

The third limitation concerns the generalizability and external validity of the findings. The present study relied on purchase intention as the primary outcome measure and recruited participants through MTurk. Specifically, this study employed purchase intention as a proxy for persuasive effectiveness. Although purchase intention is a well-established predictor of consumer behavior and a commonly used outcome measure in influencer marketing research, it does not necessarily translate into actual purchase behavior. Future studies could strengthen practical relevance by examining behavioral outcomes such as product choice, click-through behavior, or actual purchase decisions. In addition, participants were recruited through MTurk, which has been widely adopted in consumer behavior and social media research due to its efficiency and accessibility for online experiments. However, MTurk users may differ from broader consumer populations in terms of digital literacy and familiarity with online persuasive content. Therefore, future studies should replicate these findings using more diverse and representative samples.

## 6. Conclusions

This study examined how influencer image types shape consumers’ persuasion processes and purchase intentions in social media environments. Drawing on self-reference theory, ELM, PKM, and PSR literature, the findings suggest that different image formats are associated with distinct persuasion outcomes, partly because they may create attentional conditions that facilitate self-referential processing and shape persuasion knowledge activation. Specifically, brand selfies and packshots were associated with more favorable purchase intentions than consumer selfies. Persuasion knowledge partially mediated the relationship between image type and purchase intention, and higher levels of PSR weakened the negative effect of persuasion knowledge on purchase intention. Overall, this study extends PKM to visually oriented social media contexts and integrates cognitive persuasion mechanisms with relational processes in influencer marketing. The findings further suggest that effective influencer marketing depends not only on product exposure or engagement generation, but also on how visual content shapes consumers’ interpretation of persuasive intent and relational context toward influencers.

## Figures and Tables

**Table 1:** Results of measurement reliability and convergent validity assessment.

Construct	Description	Loading	Cronbach’s Alpha	CR	AVE
Persuasion knowledge (Mean = 4.820)	The way this Instagram post image that Kate sent tries to persuade people seems acceptable to me (reverse coding)	0.830	0.917	0.917	0.614
	Kate tries to manipulate the audience in ways that I don’t like.	0.806			
	I am annoyed by this post because Kate seemed to be trying to inappropriately manage or control the audience.	0.752			
	The post that Kate sent was fair in what was said and shown (reverse coding).	0.777			
	When I see the Instagram post image that Kate sent, I think it’s pretty obvious the post is trying to persuade me to buy the Herbalife’s protein powder.	0.811			
	I noticed tricks in this post image that Kate sent to promote the Herbalife’s protein powder.	0.721			
	This post image that Kate sent is meant to sell the Herbalife’s protein powder.	0.783			
PSR (Mean = 5.017)	I feel close enough to Kate to use her Instagram.	0.767	0.885	0.885	0.562
	I feel comfortable about Kate’s message on her Instagram.	0.729			
	I feel Kate is fascinating on her Instagram.	0.757			
	I think that Kate’s Instagram is helpful for my interests in health.	0.733			
	I can rely on information I get from Kate.	0.716			
	In the past, I pitied Kate when she made a mistake on her Instagram.	0.792			
Purchase intention (Mean = 4.941)	I would like to try the Herbalife’s protein powder in the post image.	0.747	0.848	0.851	0.657
	I have the intention to buy the Herbalife’s protein powder in the post image.	0.850			
	I am interested to know more where to buy the Herbalife’s protein powder in the post image.	0.830			

**Table 2:** Assessment of discriminant validity using the Fornell–Larcker criterion.

Construct	Persuasion Knowledge	PSR	Purchase Intention
Persuasion knowledge	**0.784**		
PSR	−0.142	**0.750**	
Purchase intention	−0.641	0.452	**0.811**

**Table 3:** Discriminant validity based on the heterotrait–monotrait ratio (HTMT).

Constructs	HTMT
Persuasion knowledge–Purchase intention	0.654
PSR–Purchase intention	0.451
Persuasion knowledge–PSR	0.142

**Table 4:** Assessment of common method bias using Harman’s single-factor test.

Test	Result
KMO	0.928
Bartlett’s χ^2^	4123.019
*df*	120
*p*	<0.001
Variance explained by the first factor	38.721%

**Table 5:** Descriptive statistics of main variables across conditions.

	Packshot (*N* = 152)	Brand Selfie (*N* = 153)	Consumer Selfie (*N* = 155)	Total (*N* = 460)
	*M* (*SD*)	*M* (*SD*)	*M* (*SD*)	*M* (*SD*)
Persuasion knowledge	4.98 (1.23)	3.96 (1.23)	5.51 (1.03)	4.82 (1.33)
PSR	5.01 (1.18)	5.16 (1.06)	4.88 (1.33)	5.02 (1.20)
Purchase intention	5.28 (1.25)	5.58 (1.04)	3.98 (1.50)	4.94 (1.45)
Attention to Herbalife’s protein powder	5.62 (1.02)	5.75 (1.03)	5.31 (1.37)	5.56 (1.16)
Prominent of the Herbalife’s protein powder	5.70 (0.99)	5.78 (1.05)	5.34 (1.32)	5.61 (1.15)
Brand familiarity	5.48 (1.27)	4.84 (1.70)	4.10 (1.95)	4.80 (1.76)
Attitude toward the brand	5.19 (1.23)	4.78 (1.80)	3.84 (1.77)	4.60 (1.72)
Frequency of health supplement purchases	2.70 (0.76)	2.78 (0.81)	3.00 (0.78)	2.83 (0.79)

**Table 6:** Levene’s test and Welch’s robust ANOVA results.

Dependent Variable	Levene’s *F*	*p*	Welch’s *F*	*p*
Persuasion knowledge	6.741	0.001	71.932	<0.001
Purchase intention	13.992	<0.001	60.544	<0.001

**Table 7:** Summary of hypothesis testing.

Hypothesis	Relationship Tested	Test Result
H1a	Brand selfie > Consumer selfie (Purchase intention)	Supported
H1b	Brand selfie > Packshot (Purchase intention)	Not supported
H1c	Packshot > Consumer selfie (Purchase intention)	Supported
H2a	Image type → Persuasion knowledge	Supported
H2b	Persuasion knowledge → Purchase intention	Supported
H2c	Image type → Persuasion knowledge → Purchase intention (mediation)	Supported
H3	Moderated mediation: Image type → Persuasion knowledge → Purchase intention (moderated by PSR)	Supported

**Figure A1:**
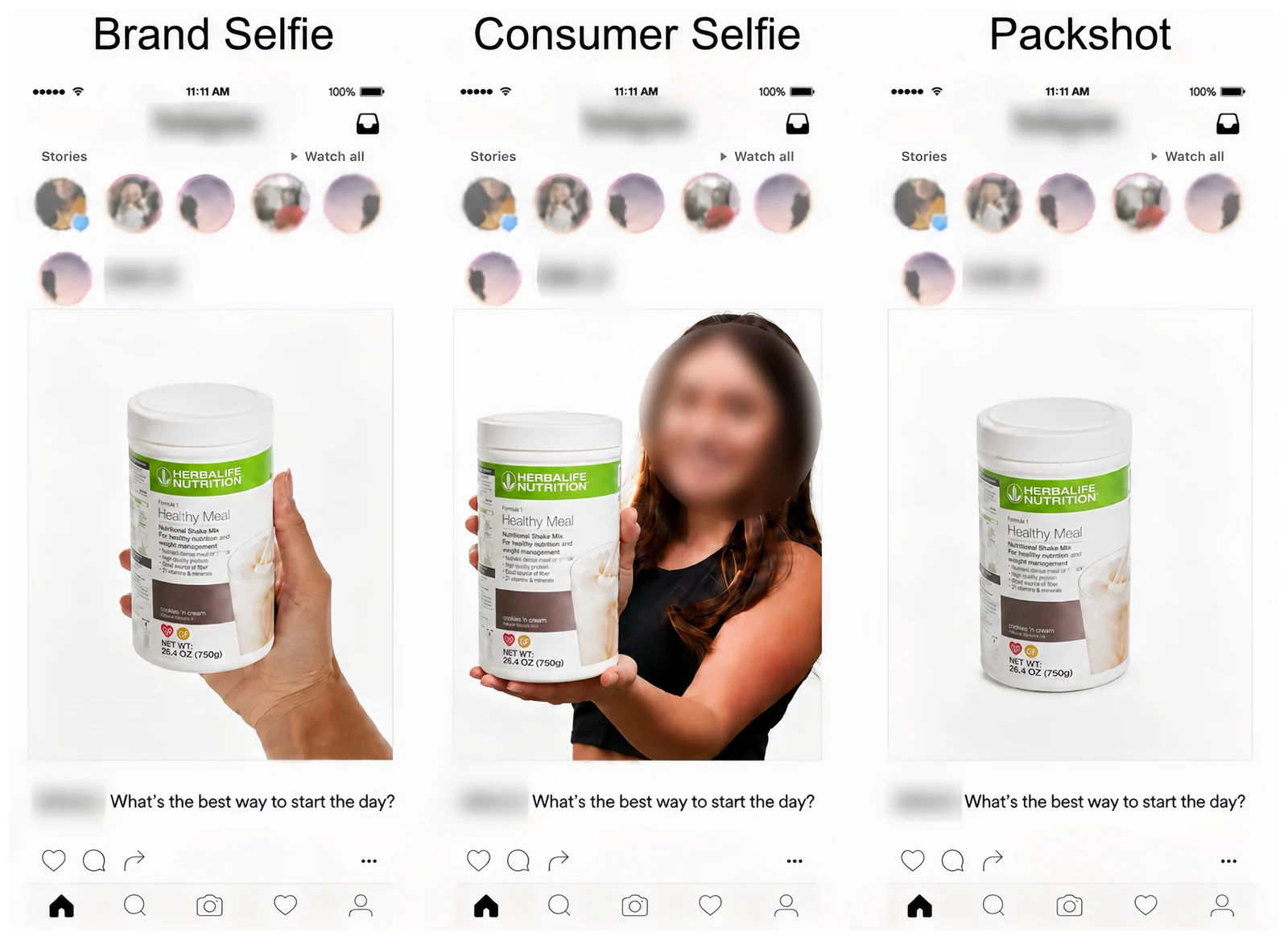
Controlled stimuli for the lab experiment. *Note:* Faces, user IDs, and platform-identifying elements in the stimulus images were blurred or partially removed in the published manuscript to protect privacy and avoid potential copyright or trademark concerns.

## Data Availability

The data presented in this study are available on request from the corresponding author due to participant privacy and ethical considerations.
